# Modified Subcuticular Stitch

**DOI:** 10.4103/0974-2077.44173

**Published:** 2008

**Authors:** George Alex

**Affiliations:** *Department of Breast Surgery, Castle Hill Hospital, Castle Road, Cottingham HU16 5JQ, United Kingdom*

## BACKGROUND

In the conventional method of putting subcuticular stitches, the subcuticular stitch is started by inserting a knot at one end of the incision. The short end of the suture is cut. A small bite is taken of the subcuticular material and the suture is pulled through. Then on the opposite side of the wound a similar subcuticular bite of the suturing material is inserted and gently worked up the wound.[1]

A problem faced in this procedure is that the knotted wound edge keeps getting pulled into the wound while taking subcuticular bites. We describe a method to over come this problem.

## METHOD

**Figure 1:**
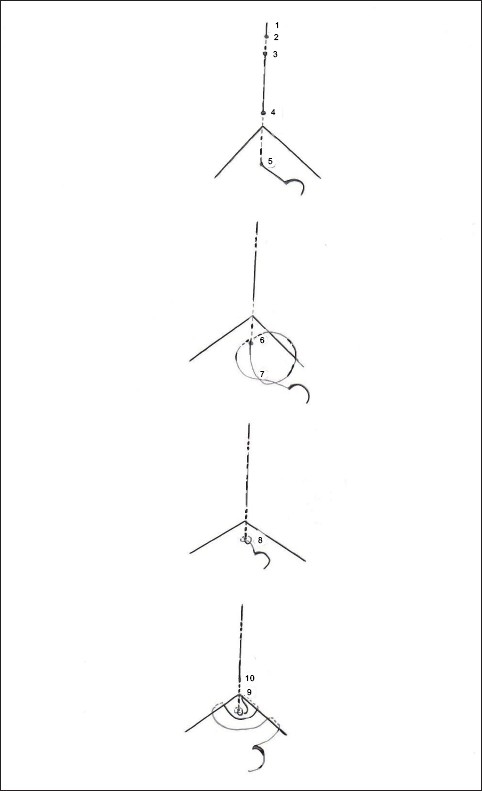
Modified subcuticular stitch

A modified subcuticular stitch is described here [Figure 1]. A few cm from one wound edge the suture needle is passed through the skin (2) and brought out again (3). Almost the full length of suture is pulled through and a small artery clip applied at its end (1). A few mm away from the distal end of the wound (4), the suture needle is passed through the skin into the wound through the dermal layer (5). The tip of the needle is passed into the dermal layer again (6), a loop is formed and the needle is passed through the loop (7) to form a knot (8).[2] For a more secure knot two throws may be tied at this point as in a Surgeons knot. Subsequently subcuticular stitches are applied (9) in the conventional way. When stitching has been completed the suture can be cut at the distal end of the wound (10). The artery clip with the bit of suture (1 to 4) attached to it can be removed.

It can be seen that while putting subcuticular stitches, the knot at the wound edge does not move into the wound due to the extra stability provided by this technique thereby helping in better approximation of wound edges.

## CONCLUSION

The modification described helps in better approximation of the edges.

## References

[1] Moy RL, Lee A, Zalka A (1991). Commonly used suturing techniques in skin surgery. American Family Physician.

[2] Singh-Ranger D (2003). A simple technique for the retention of a subcuticular suture. Surgeon.

